# *Lactiplantibacillus plantarum*-mediated modulation of volatile flavor and quality in low-salt spontaneously fermented yellow capsicum sauce

**DOI:** 10.1038/s41538-026-00854-z

**Published:** 2026-04-22

**Authors:** Lijuan Zhou, Dengcheng Li, Yuxin Huang, Jiamu Kang, Yuyun Lu, Lin Zhang, Shao-Quan Liu

**Affiliations:** 1 School of Food Science and Engineering, Hainan University, Haikou, China; 2 Key Laboratory of Food Nutrition and Functional Food of Hainan Province, Haikou, China; 3 Key Laboratory of Tropical Agricultural Products Processing Technology of Haikou, Haikou, China; 4 Department of Food Science and Technology, National University of Singapore, Singapore, Singapore

**Keywords:** Biotechnology, Microbiology

## Abstract

Yellow capsicum sauce (YCS) is a special fermented condiment in Hainan province, China, and its fermentation typically occurs in a high-salt environment. In this study, the effects of different salt contents (5, 10, 15, and 20%, w/w) on microbial communities and volatile flavor profiles in YCS were systematically investigated by metagenomic approach and HS-SPME-GC-MS. The results revealed that *Lactiplantibacillus* (54.66%) was the dominant genus in low-salt samples (SF5), while its abundance was less than 6% in higher salinity levels (SF15 and SF20). A total of 48 volatile flavor compounds (VFCs) were detected in the naturally fermented YCS, with alcohols and esters being the primary VFCs. Low-salt fermentation facilitated the accumulation of VFCs, and the total VFCs content in SF5 was the highest. Aroma compounds showed a strong correlation with *Lactiplantibacillus plantarum*. To further validate the findings, *L. plantarum* MA1 isolated from SF5 was inoculated into the low-salt YCS substrate for bioaugmented fermentation. This strain significantly increased key aroma components, such as *cis*-3-hexenyl isovalerate, hexyl 3-methylbutanoate, and ethyl acetate. Moreover, it significantly increased the lactic acid content while reducing the nitrite content, thereby more effectively preserving the fresh yellow color of capsicum sauce and the stability of its spiciness.

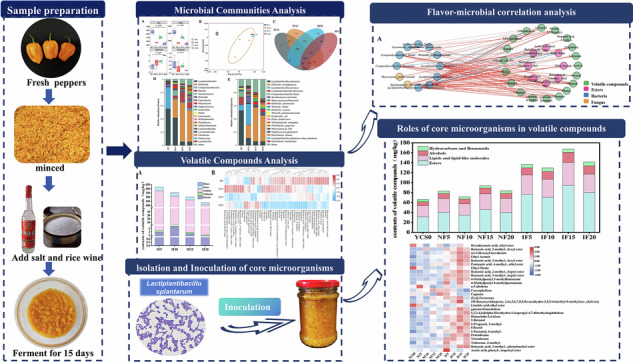

## Introduction

*Capsicum chinense* (also known as habanero-type pepper) is a kind of yellow chili pepper unique to Hainan province in China. The shape of this pepper resembles a lantern, and it possesses a potent spiciness level of up to 150,000 Scoville Heat Units (SHU)^1^. The capsicum is not usually eaten directly, but is pickled and fermented to make a hot sauce. Yellow capsicum sauce (YCS) is a condiment popular in Hainan Province, which is famous for its unique spicy taste and distinctive local characteristics. This sauce not only enriches the local food culture, but also becomes one of the important economic products.

The unique flavor and texture of YCS mainly result from complex microbial fermentation processes, such as lactic acid bacteria (LAB), which can convert sugar into lactic acid, endowing YCS with its characteristic sour and hot taste^2,3^. However, there are various LAB, and their types change in different stages of fermentation^4^. Besides LAB, there are several other microorganisms, such as yeasts and acetic acid bacteria (AAB), which also play a certain role in promoting the flavor of YCS. Yeast can ferment glucose to generate ethanol and carbon dioxide, but also produce glycerol, propanol, isobutanol, active amyl alcohol, and other alcohols^5^. AAB can make acetic acid, giving food a sour odor, and enhancing food’s flavor by using alcohol in food for oxidative fermentation^6^. In general, these microorganisms exert a pivotal effect on the flavor of YCS by synthesizing a diversity of enzymes and metabolites. They collaboratively engage in the fermentation process of YCS, conferring on it a distinctive and ample flavor^7,8^.

The biosynthesis and accumulation of distinctive flavor substances in YCS are mainly dominated by the metabolic activity of the microbial community. The salt content, as an important control parameter in the fermentation process, exerts a notable influence on the fermentation speed, direction, and microbial community structure^9^. Salt not only inhibits the proliferation of deleterious microorganisms, such as spoilage and pathogenic microorganisms, but also influences the activity of advantageous fermentative microorganisms, such as LAB and yeasts, thereby regulating the flavor and quality of fermented products^10^. The traditional procedure of YCS production involves removing the stems from the capsicum, chopping them finely, mixing them with salt and rice wine, and then placing them into jars to ferment for about 2 weeks. The open preparation procedure in this process leads to inconsistent flavor and quality. The product is susceptible to browning, which greatly impedes the development of the industry. At the same time, the addition of salt exceeds 10% (w/w), which is not conducive to the growth of beneficial microorganisms, such as LAB. When the salinity exceeds their tolerance range, the growth and metabolic activities of LAB are inhibited, which may give rise to insufficient acidity of YCS, thereby affecting its flavor. Additionally, the high salt content will also exert adverse effects on the health of consumers. Therefore, in the production of YCS, controlling the appropriate salinity is crucial to ensure the optimal growth of LAB, the quality properties of fermented products and the well-being of consumers.

The application of multi-omics technology in elucidating flavor formation in fermented products has achieved remarkable progress^11^. This technique has extensive applications in the investigation of functional microorganisms that drive the flavor formation of products, involving multiple levels such as genomics, transcriptomics, proteomics, and metabolomics^12,13^. The utilization of multi-omics technology enables a profound understanding of the physiological characteristics and ecological functions of microbiome, and allows for the exploration of how microbial communities affect the development of flavor compounds. The application of these new technologies will contribute to providing more reliable data-backed evidence for the research and utilization in related fields^14^. However, the flavor substances in fermented products are derived from various metabolic products of the microbial community. The flavor of YCS is the outcome of the fermentation by a variety of tropical microorganisms in Hainan Province. The succession of microbial communities and variability of the fermentation environment give rise to the extremely complex flavor formation mechanisms^15^. So far, the mechanism of microbiota regulating the generation of flavor substances has not been elucidated, especially the impact of microbial metabolic activities on the volatile flavor compounds (VFCs) profile of YCS is not yet fully understood.

We hypothesize that salt concentration modulates microbial community composition and functional metabolism, thereby determining the flavor profile of YCS. Moreover, *L. plantarum* is a core functional bacterium in low-salt fermentation of YCS, directly contributing to the biosynthesis of VFCs and consequently influencing overall product quality. Therefore, omics approaches were used to characterize the microbial community and the distinctive VFCs profile of YCS produced under different salt levels, and to elucidate how salt regulates flavor development through microbial metabolic activities. Based on these results, a low-salt fermentation system was established and inoculated with core functional strains (*L. plantarum*) to validate strain–flavor relationships. This work is expected to improve the quality and safety of traditional capsicum sauce and to provide a scientific basis for quality control and innovation in other traditional fermented foods.

## Results and discussion

### α-Diversity and β-diversity analysis

**Fig. 1:**
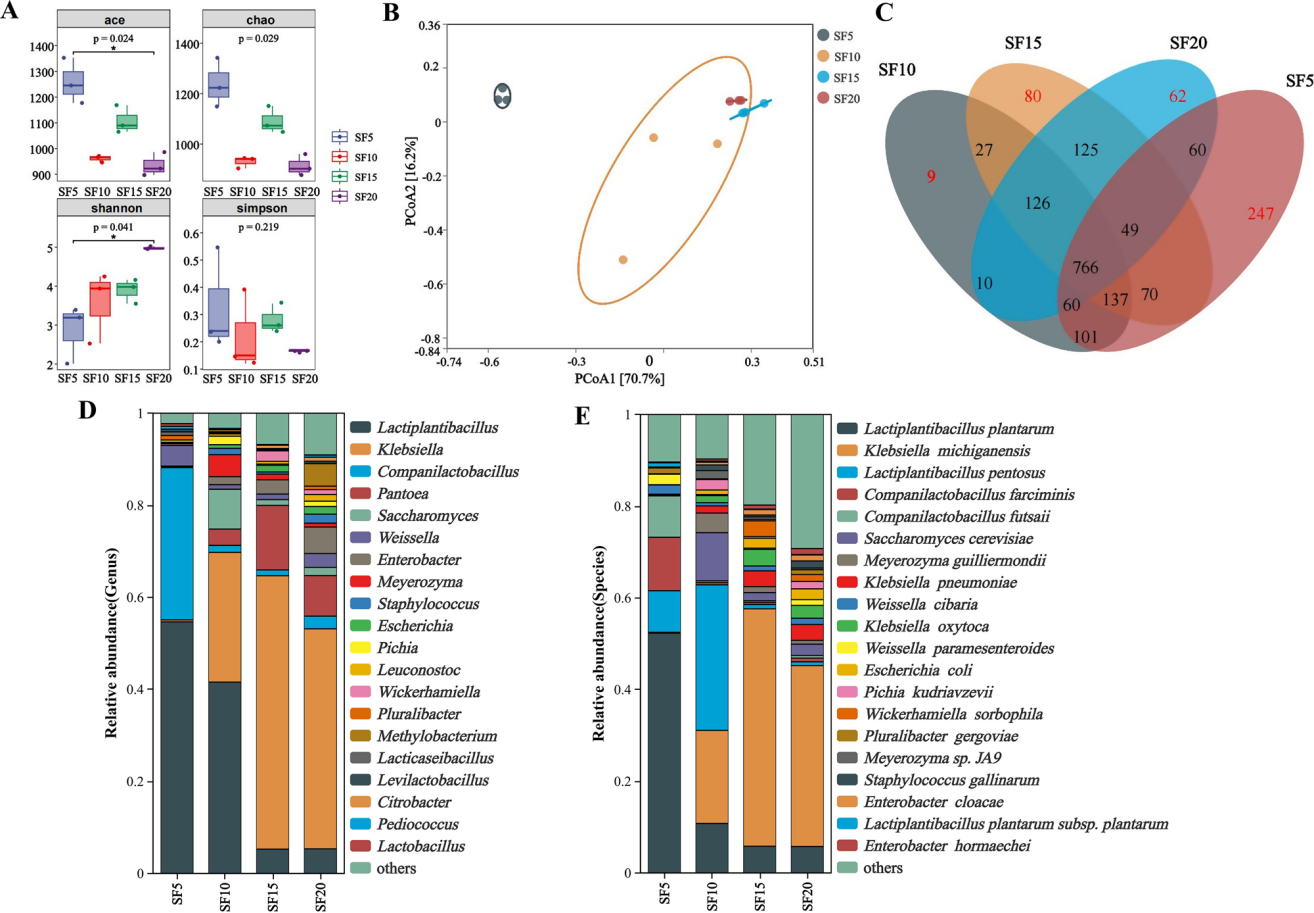
Microbiome analysis of YCS with different salt levels. Alpha diversity (**A**) was estimated using ACE, Chao1, Shannon and Simpson index. Beta diversity (**B**) was presented using principal coordinates analysis (PCoA) score plot based on Bray-Curtis matrix. (**C**): Venn diagram of species counting. (**D**): Composition and relative abundance of genus. (**E**): Composition and relative abundance of species. (Source: Microsoft Office PowerPoint, GenesCloud platform, Majorbio Cloud Platform).

The alpha diversity index is used to assess the richness and evenness of a community^16^. Among the four salinity samples, SF5 exhibited the highest average ACE index and Chao index values of 1258.51 and 1238.23, respectively, indicating that SF5 contains a greater number of species than the other groups (Fig. 1A). In contrast, the average ACE index and Chao index of SF20 samples were the lowest, merely 935.22 and 913.13, respectively. It indicates that salinity has a significant impact on the species richness of YCS. The Shannon index and Simpson index were used to measure the richness and evenness of a microbial community. A higher Shannon index indicates greater species evenness, whereas a higher Simpson index indicates lower species evenness. Further comparison of the sample Shannon index and Simpson index of the four groups showed that the average Shannon index of SF20 was the highest (4.98), while the average Simpson index of SF20 was the lowest (0.166), which was significantly different from that of SF5, SF10, and SF15. This result reveals that the community evenness in the SF20 samples was notably greater than that in the other three treatment groups.

The positions of the four treatments in the PCA space differed significantly, indicating substantial diversity differences among them (Fig. 1B). The SF5 group was far from the SF10, SF15, and SF20 groups, indicating a low degree of community similarity between the SF5 group and the other three treatment groups.

### Microbial annotation

A Venn diagram was used to show the distribution of species across the four treatment groups. As shown in Fig. 1C, the four groups shared a total of 766 species. The SF5 samples had the highest number of unique microbial species, reaching 247 species. This finding suggests that low salinity provides a suitable environment for certain microorganisms and may not be high enough to inhibit the growth of most microorganisms, nor does it result in excessive simplification of the microbial population in capsicum sauce. The SF10 samples had 9 unique microbial species, indicating that this salinity level imposes intense environmental selection pressures. Most salt-sensitive microorganisms struggle to survive due to hyperosmotic stress. At salinities of 15% (SF15) and 20% (SF20), the number of endemic species was 80 and 62, respectively. This may be related to the enrichment and dominance of specific halotolerant microorganisms (halophiles/halotolerants) under these conditions. At the genus level (Fig. 1D), the dominant genera of SF5 were *Lactiplantibacillus* and *Companilactobacillus*, with their relative abundances being 54.66% and 33.10%, respectively. The main dominant genera of SF10 included *Lactiplantibacillus* and *Klebsiella*, with relative abundances of 41.55% and 28.20%, respectively. However, the structure of the microbial community changed significantly in SF15 and SF20, with the dominant genus changing to *Klebsiella*, and relative abundances being 59.41% and 47.80%, respectively. This might be attributed to the high salt tolerance of *Klebsiella*, enabling them to survive in environments with a higher salinity^17,18^. In addition, the relative abundances of fungi in the SF5, SF10, SF15, and SF20 groups were 0.23%, 15.86%, 8.24%, and 7.03%, respectively, which were markedly lower than those of bacteria. This may be related to the high-temperature and high-humidity conditions of the tropical Hainan region, which favor bacterial growth, particularly lactic acid bacteria. For example, in traditional fermented foods from Hainan, including fermented vegetables, fermented fish tea, and fermented grains, lactic acid bacteria are generally the predominant microorganisms^19^.

At the species level (Fig. 1E), the dominant species in the low-salinity group (SF5) was *L. plantarum*, which had a relative abundance of 52.30%. It was significantly higher than that of other species, indicating that this low salinity condition provides a favorable environment for the survival of *L. plantarum*. This was followed by *C. farciminis* with a relative abundance of 11.66%, which is commonly associated with beneficial effects in food fermentation process, such as extending the shelf life of the food and enhancing its flavor. When the salt content increased to 10%, *L. pentosus* became the dominant species in SF10, with relative abundance increasing to 31.75%. While the relative abundance of *Klebsiella michiganensis* was 20.29%, which began to show its salt-tolerant characteristics. In the higher salinity groups (SF15 and SF20), the dominant species was *Klebsiella michiganensis*, with relative abundances of 51.78% and 39.47%, respectively. These results indicate that *K. michiganensis* can survive in high-salinity environments. This species has been reported as an opportunistic pathogen and may carry genes associated with antibiotic resistance^20^. In addition, *Klebsiella pneumoniae* and *Klebsiella oxytoca* were detected in YCS, though both species constituted less than 3.5% of the total bacterial community. Both species are Gram-negative, non-motile bacilli, widely distributed in the environment, and can be part of the human gut microbiota. As opportunistic pathogens, they may pose potential safety risks under specific conditions^21^.

Compared to long-term fermented Hunan peppers, although both exhibit lactic acid bacteria dominance under low-salt or early-stage fermentation, with an increased proportion of salt-tolerant or opportunistic pathogens during high-salt or long-term fermentation, there are significant differences in the dominant fungi: the main fungus in Hainan’s YCS is *Saccharomyces*, while fermented red peppers in Henan are dominated by *Zygosaccharomyces rouxii*^22^. This difference in fungal composition may have a certain impact on the flavor formation of the products. In our study, Hainan chili sauce fermented at 10% (w/w) salt was dominated by *Lactiplantibacillus*, whereas under the same salt conditions, *Rosenbergiella* was the dominant bacterial genus in Guizhou chili sauce^23^. This suggests that differences in raw materials or geographic origin may affect the composition of microbial communities in fermented chili.

The above findings revealed the significant alterations of microbial communities in capsicum sauce under different salinity conditions, indicating that the microbial population could be effectively controlled by precise control of salinity, thereby improving product safety and enhancing flavor. The low-salt fermentation significantly promoted microbial abundance and diversity, and the dominant bacterial communities (such as *L. plantarum*) that had quality improvement functions showed an enrichment advantage. As the core functional strain, *L. plantarum* produces lactic acid and other metabolites by metabolizing carbon sources such as lactose, and this biological characteristic has been confirmed by research^24,25^. Its metabolic activity effectively reduces the pH, inhibits the proliferation of undesirable microorganisms, and extends the product’s shelf life. Simultaneously, this strain generates multiple flavor compounds—including alcohols, ketones, and esters—that significantly enhance the sensory properties of fermented foods. Previous studies confirm that these volatiles are key to shaping product aroma intensity^26,27^. In addition, *L. plantarum* also has a certain probiotic function (strain-dependent), which can regulate the balance of human intestinal microbiome and promote digestive health^28,29^. Therefore, the condition of low salinity is not only beneficial to the growth and metabolism of *L. plantarum* but also can significantly improve the quality and safety of food through its fermentation, bringing many benefits to the food industry^30^.

### Volatile compounds of spontaneously fermented YCS with differing salt contents

The aroma compounds characteristics of YCS under different salinity conditions were determined by HS-SPME-GC-MS analysis, with the results detailed in Table S1. Altogether, 48 volatile compounds were identified, indicating that the aroma compounds composition of capsicum sauce was complex. Among the compounds detected, esters (18) and alcohols (15) were the most abundant, and these two classes of compounds played a key role in the formation of YCS flavor. Esters typically possess a fruity or floral aroma profile, while higher alcohols may contribute sweetness at high concentrations. Apart from esters and alcohols, ketones (6), aldehydes (5), carboxylic acids (2), and terpenoids (1) were also detected. These compounds, despite being present in relatively smaller amounts, could still contribute to the overall aroma. Ketones and aldehydes conferred a strong herbal aroma, and terpenoids endowed YCS with special spice aroma characteristics, while carboxylic acids exhibited a rancid odor.

**Fig. 2:**
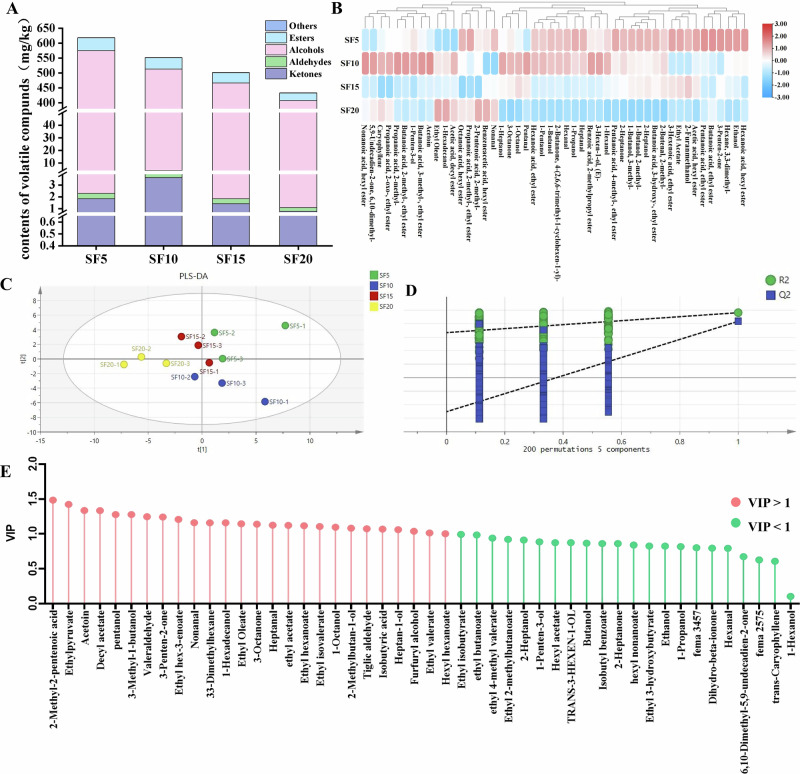
Volatile compounds of YCS identified by HS-SPME-GC-MS. **A** The total content of all volatile compounds (mg/kg) in YCS with different salt contents. **B** Heatmap of volatile compound content. The ordinate represents the grouping of different samples, and the Abscissa represents the 48 volatile compounds. The bubble size represents the size of the value. Red indicates the high expression of the substance content. Blue indicates that the substance content is low. **C** PLS-DA score plot. **D** Model cross-validation results. **E** VIP value of volatile compounds. (Source: Microsoft Office PowerPoint, Origin, TBtools, SIMCA, GraphPad Prism).

The content of VFCs in YCS was analyzed (Fig. 2A), and it was found that the higher the salinity, the lower the total content of VFCs. The total content of VFCs in SF5 was the highest (618.75 mg/kg), followed by SF10 (552.11 mg/kg), while the total content of VFCs in SF15 and SF20 with higher salinity was less than 502 mg/kg. These results indicate that a 5% salt content was more conducive to the production of VFCs in YCS. In addition, in terms of volatile flavor substance categories, esters and alcohols were the most abundant among the identified VFCs.

Figure 2B presents a heatmap constructed based on the concentrations of VFCs, which was used to visually illustrate the distribution patterns of individual VFCs under different salinity conditions. As clearly shown in the figure, distinct differences in the composition of VFCs were observed among the different salinity treatment groups, indicating that salt concentration had a significant impact on the volatile flavor profile of YCS.

Overall, most esters and alcohols exhibited higher concentrations in the low-salt samples (SF5 and SF10), whereas their concentrations were lower in the high-salt samples (SF15 and SF20). For example, key VFCs such as ethyl acetate, ethyl hexanoate, ethyl isovalerate, and 1-hexanol were present at higher levels in SF5 and SF10, but at lower levels in SF15 and SF20. This trend was consistent with the variation observed in the total content of VFCs.

VFCs in YCS samples with different salinity levels were analyzed using Partial Least Squares Discriminant Analysis (PLS-DA) to evaluate the differences among groups. As shown in the PLS-DA score plots (R²X = 0.852, R2Y = 0.939, Q² = 0.663; Fig. 2C, D), samples from SF5, SF10, SF15, and SF20 exhibited a certain degree of separation, indicating that salinity influenced the volatile flavor profiles of YCS. To validate the robustness of the model, a permutation test was performed, yielding an R^2^ intercept of 0.303 and a Q² intercept of −0.473, suggesting that the model was not overfitted. A total of 26 biomarkers of YCS (Fig. 2E) were screened through VIP values > 1, including 7 alcohols, 9 esters, 3 ketones, 4 aldehydes, and 3 other compounds. Among them, ethyl acetate, hexyl acetate, ethyl hexanoate, and ethyl isovalerate were important aromatic compounds in YCS, which impart pineapple, apple, grape, and banana flavors to YCS. Meanwhile, ethyl hexanoate gives YCS brandy flavor. The odorant, 1-hexanol, endows YCS with floral, fruity, grassy, and other aromas. 2-Methylbutan-1-ol can bring rich fish oil, malt, onion, and wine flavor characteristics to YCS. 3-Methyl-1-butanol adds caramel, cocoa, floral, and malt aromas, and pentanol adds vanilla, fruit, and nutty aromas to YCS, and 2-heptanol adds citrus, mushroom, and oil flavors to YCS.

The content of aroma compounds in YSC varied with salt concentration. For instance, ethyl isovalerate, ethyl hexanoate, and 1-hexanol exhibited higher concentrations in SF5 and SF10 samples, whereas their concentrations were lower in SF15 and SF20 samples. Additionally, the concentrations of ethyl acetate and hexyl acetate—two key ester compounds—were significantly higher in SF5 compared to those in other salinity groups. This indicates that under low salt concentration conditions, microbial metabolic activities may be more vigorous, promoting the formation of these aromatic compounds. In contrast, under high salinity conditions, microbial metabolism may be more inhibited, leading to reduced concentrations of these aroma compounds. The above results demonstrate that salinity significantly influences the content of key aroma compounds in YCS, providing important reference data for capsicum sauce production and quality control.

### Roles of microorganisms in flavor formation

**Fig. 3:**
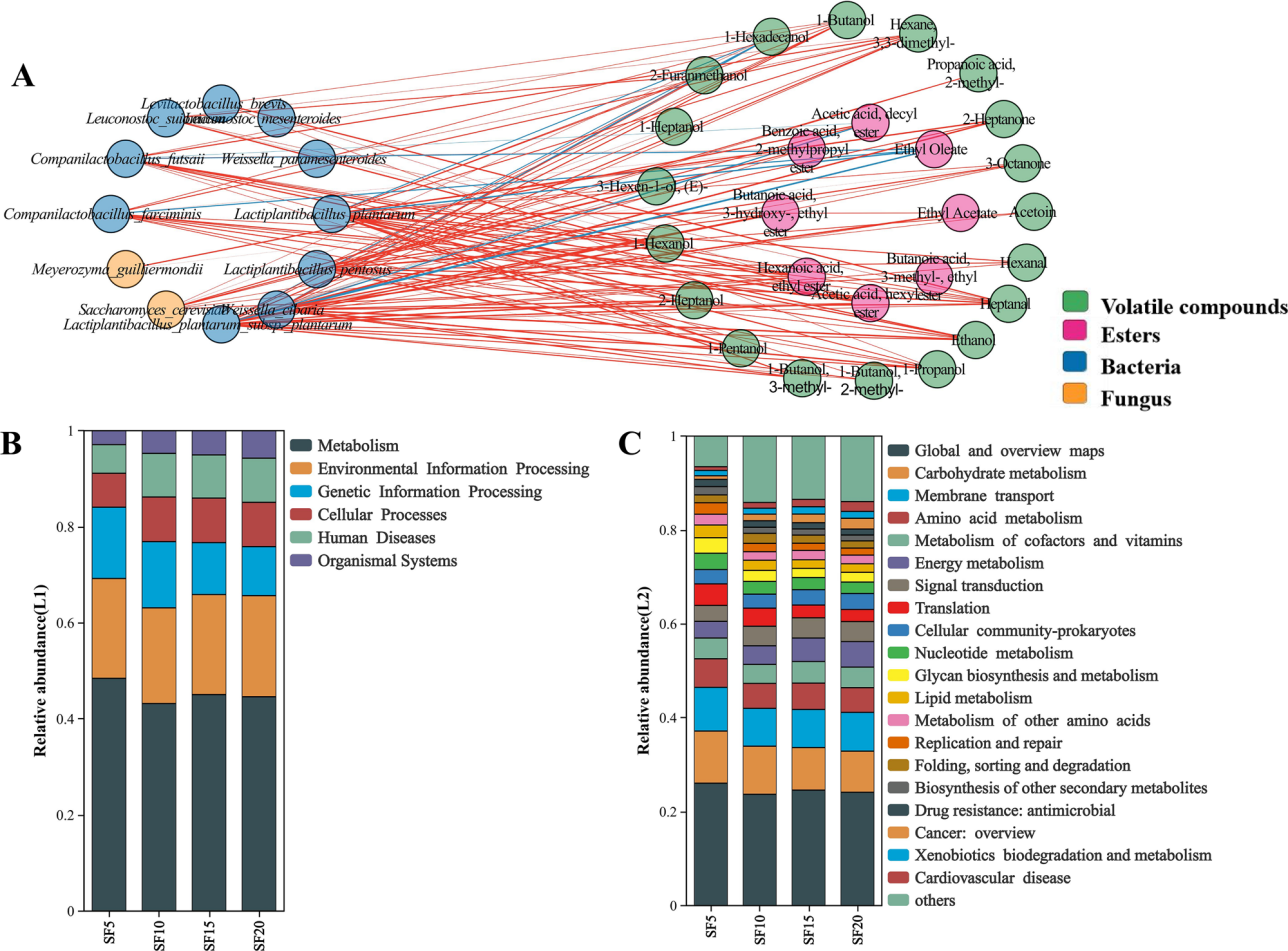
Spearman correlation network and KEGG pathway analysis of YCS under different salt contents. Spearman correlation network (**A**) among volatile compounds and species in YCS with different salt contents. The blue nodes on the left represent bacterial species names, while the pink nodes denote fungal species names. The purple nodes on the right represent ester compound names, and the green nodes denote other types of compound names. The red line represents the positive correlation. The blue line represents a negative correlation. The thickness of the line represents the strength of the correlation. The thicker the line indicates the stronger the correlation. **B** KEGG primary metabolic pathway analysis. **C** KEGG secondary metabolic pathway analysis. (Source: Microsoft Office PowerPoint, Cytoscape, Majorbio Cloud Platform).

Correlation analysis of microbial species and volatile compounds revealed that 27 volatile compounds in YCS showed significant correlations (|*R* | > 0.5) with 12 species (Fig. 3A). *L. plantarum* exhibited positive correlations with 5 esters, including ethyl acetate (*R* = 0.57), hexyl acetate (*R* = 0.67), ethyl hexanoate (*R* = 0.62), ethyl 3-hydroxybutyrate (*R* = 0.71), and isobutyl benzoate (*R* = 0.55). In addition, *L. pentosus* showed positive correlations with ethyl hexanoate (*R* = 0.63), ethyl 3-hydroxybutyrate (*R* = 0.61), and isobutyl benzoate (*R* = 0.63). Further, *C. farciminis* and *C. futsaii* exhibited a positive correlation with hexyl acetate, ethyl hexanoate, isobutyl benzoate, and 1-hexanol. Although numerous positive correlations were found, negative correlations also existed between volatile compounds and core species. For example, decyl acetate had a negative relationship with *W. cibari* (*R* = − 0.63) and *C. futsai* (*R* = − 0.52). In the study by Liu et al. (2024), the dominant fungal genera in chili sauce from Hebei Province were *Zygosaccharomyces*, *Pichia*, *Saccharomyces, Starmerella*, and *Debaryomyces*, with fungal abundance being notably high. Among them, *Pichia* was highly positively correlated with multiple organic acids and VFCs, representing one of the key functional microbes contributing to chili sauce flavor formation^31^. In contrast, in our study, bacterial relative abundance in YCS was much higher than that of fungi and was closely associated with the formation of key VFCs.

These correlation analysis findings reveal the complex microbial metabolic network in YCS, where certain bacteria might be associated with the production of specific flavor substances. Understanding these interactions is essential for optimizing the capsicum sauce production process and enhancing the final product’s flavor quality and consistency. By regulating the microbial community during the production process, the taste of the product and the overall consumer experience can be ameliorated.

KEGG is a widely utilized bioinformatics resource dedicated to resolving the advanced functions of genes and related biochemical networks in living organisms. The functional genes in YCS were predicted by comparing the sequenced genes with the KEGG database. As shown in Fig. 3B, C, there were significant differences in the number of annotated genes in YCS with different salinity levels. Samples with lower salinity had the most annotated genes, while those with higher salinity contained fewer annotated genes. At the primary taxonomic level (Fig. 3B), the metabolic functions of all samples were dominated by six core pathways, including metabolism, environmental information processing, and genetic information processing. The most abundant pathways in the four groups of samples were metabolism-related genes, followed by environmental information processing, whereas the genes related to organismal systems were the fewest.

At the secondary taxonomic level (Fig. 3C), the top 15 KEGG functionally annotated genes with relative abundances were analyzed in depth. Global and overview maps (26.08%), carbohydrate metabolism (11.09%), membrane transport (9.32%), and amino acid metabolism (6.08%) were the four main pathways in SF5. Although the other samples with higher salinity were also dominated by these four pathways, their relative abundance was lower than that of SF5. It can be inferred that the flavor formation of YCS may primarily rely on global maps and carbohydrates as the essential building blocks.

In terms of carbohydrate metabolism, LAB efficiently decomposed sugars in peppers through homo- and/or heterofermentation to produce lactic acid (dominant acidity) and some pyruvate (key intermediate metabolite), which can be further converted into acetyl-CoA. These provided precursors for the synthesis of esters (such as ethyl acetate) and imparted capsicum sauce with fruity flavor. In membrane transport, active transmembrane material exchange promoted the uptake of substrates such as sugars and amino acids and the secretion of metabolites, and accelerated the accumulation of lactic acid and the generation of flavor precursors. In amino acid metabolism, LAB converted amino acids into volatile aldehydes and ketones (such as valeraldehyde, hexanal, dihydro-beta-ionone) through deamination or transamination. These substances might be synergized with alcohols and esters to form complex floral, fruity, and malty aromas. Under low-salt conditions, LAB effectively utilize carbohydrates through the aforementioned metabolic pathways, promoting lactic acid production and the accumulation of flavor precursors. This significantly enhanced the aromatic complexity and shelf life of capsicum sauce. Conversely, high-salt environments inhibited the activity of these metabolic pathways, resulting in a monotonous flavor profile.

Based on previous research, *L. plantarum* was identified as a core microorganism in low-salt samples and is closely associated with VFCs in YCS. To investigate the relationship between this strain and flavor formation, we subsequently conducted inoculated fermentation experiments.

### Changes in acidity during fermentation of YCS with *L. plantarum*

**Fig. 4:**
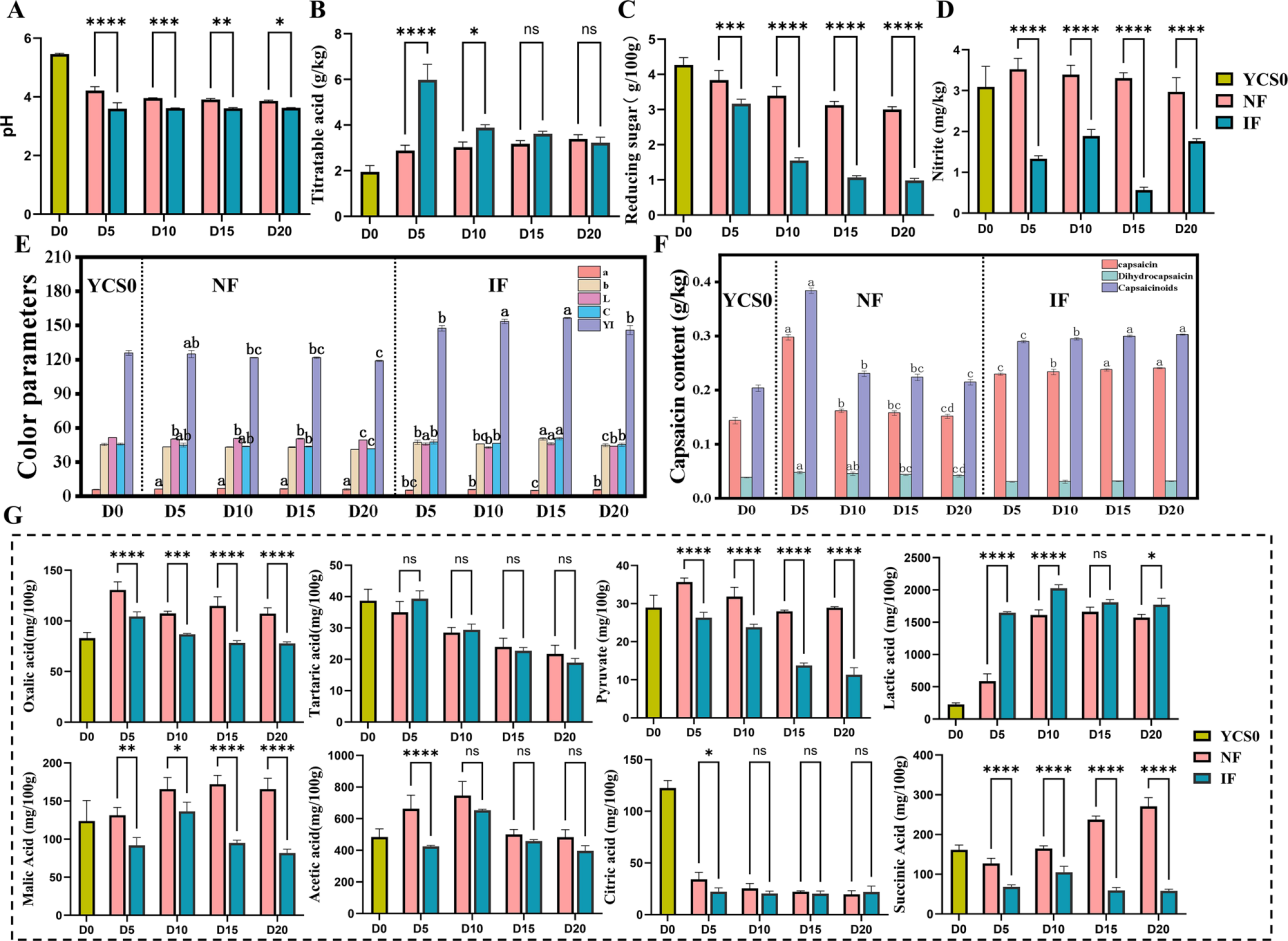
The physicochemical properties of YCS during spontaneous fermentation (NF) and Lactiplantibacillus plantarum MA1-fortified fermentation (IF). **A** pH value. **B** Titratable acid content. **C** Reducing sugar content. **D** Nitrite content. **E** Color parameter analysis. **F** Capsaicin content. **G** Organic acid content. Note: The level of significance is indicated by alphabetical or asterisk markings (ns; *p* > 0.05; *p < 0.05; **p < 0.01; ***p < 0.001). The different lowercase letters in (**E**, **F**) indicate statistically significant differences in color parameters and capsaicin content at different fermentation time points (D5–D20) (*p* < 0.05, *n* = 4). (Source: Microsoft Office PowerPoint, GraphPad Prism, Origin).

pH and total acidity are key physicochemical indicators for evaluating fermented foods, and their changes can indirectly reflect the intensity of microbial metabolic activity. As shown in Fig. 4A, the pH of NF decreased to approximately 4.2 on day 5, whereas the pH of IF decreased further to 3.8. From day 10 to 20 of fermentation, the pH of both groups of samples eventually stabilized, with NF stabilizing between 3.8–4.0 and IF between 3.6–3.7. During the critical fermentation stage (days 5–15), IF showed more significant acid-producing characteristics, and its total acid content was consistently higher than that of NF. At the initial stage of fermentation (day 5), the total acid content in IF reached 6 g/kg (Fig. 4B), significantly higher than the 2.88 g/kg in NF.

The above differences may stem from a distinct microbial community. The inoculation of *L. plantarum* MA1 in the IF group accelerated acid accumulation, thereby lowering pH more quickly. In addition, a lower final pH also helps inhibit the growth of miscellaneous bacteria and improves the stability and controllability of the fermentation process. The findings indicated that induced fermentation had obvious advantages in improving acid production efficiency and acidification rate, which was helpful for optimizing the fermentation procedure and quality control of YCS.

### Changes in reducing sugar content during fermentation of YCS with *L. plantarum*

As shown in Fig. 4C, the content of reducing sugars in NF and IF decreased with the extension of fermentation time. The reducing sugar content of NF gradually decreased from 4.27 g/100 g to 3.00 g/100 g by day 20, indicating that the sugar consumption rate was slow and stable during fermentation. In contrast to NF, the reducing sugar content of IF decreased sharply over time. By the 20^th^ day of fermentation, it had dropped to only 0.98 g/100 g, showing a significant reduction, particularly from day 5 to day 10. This was attributed to the active metabolism of the inoculated *L. plantarum* MA1, which consumed substantial amounts of reducing sugars^32^. Consequently, the sugar utilization efficiency in the IF was notably greater than that in the NF.

### Changes in nitrite content during fermentation of YCS with *L. plantarum*

The nitrite content of NF remained relatively stable throughout the entire fermentation process (Fig. 4D), starting at 3.09 mg/kg on day 0, then increasing to 3.52 mg/kg on day 5, followed by a decrease to 2.97 mg/kg, with minimal overall fluctuations. However, the nitrite content in IF decreased rapidly to 1.33 mg/kg on day 5 and eventually reached a minimum of 0.56 mg/kg. Once fermentation began, the nitrite level in IF was consistently lower than in NF throughout the process. This indicates that inoculating with *L. plantarum* MA1 notably enhances nitrite degradation in the fermentation process, likely due to increased metabolic activity or the production of nitrite reductase^33^. In contrast, the natural fermentation group, relying solely on the metabolism of environmental microorganisms, exhibited lower nitrite removal efficiency. The induced fermentation process showed obvious advantages in controlling nitrite accumulation, especially in the key stages of fermentation (such as day 15), which can significantly reduce this substance and ensure food safety.

### Changes in chroma value during fermentation of YCS with *L. plantarum*

In this dynamic fermentation process, the a*, b*, YI, and C* values of IF were consistently higher than those of NF (Fig. 4E). The a* value of NF initially increased and then decreased, peaking at 6.83 on day 10. In contrast, the b* value declined steadily over the same period. YI is used to measure the degree of yellowness of a food, reaching its maximum on day 0 of fermentation. The YI of NF and IF reached their minimum at 145.99 and 119.00, respectively, on day 20. The C* value of NF decreased with extended fermentation time, ranging from a maximum of 48.88 to a minimum of 41.70. Compared with NF, the C* value of IF declined to 45.30 on day 20, and remained consistently higher than that of NF. The above results showed that the induced fermentation process could maintain the bright yellow color of capsicum sauce more effectively, and the stability of YI and C* values was significantly better than that of spontaneous fermentation, which may be related to the modulation of pigments by inoculated microorganisms^34^.

### Dynamic variations in capsaicin and organic acids contents during fermentation of YCS fermented with *L. plantarum*

As shown in Fig. 4F, capsaicin, dihydrocapsaicin, and total capsaicinoid contents in NF peaked on day 5, reaching 0.298 g/kg, 0.048 g/kg, and 0.384 g/kg, respectively, and then decreased significantly to 0.152 g/kg, 0.042 g/kg, and 0.215 g/kg on day 20. The corresponding changes in SHU of NF followed a similar trend, increasing from 3138.040 on day 0 to 5920.512 on day 5, and then steadily decreasing to 3322.902 on day 20 (Fig. S1). In contrast, all parameters of IF remained stable throughout fermentation. The capsaicin content of IF ranged between 0.230 and 0.241 g/kg, the dihydrocapsaicin content of IF stabilized at 0.031–0.032 g/kg, and the total capsaicinoid content of IF fluctuated minimally (0.290–0.303 g/kg). The SHU in IF correspondingly remained stable, ranging between 4469.856 and 4673.603. The findings show that fermentation with *L. plantarum* MA1 effectively inhibited the degradation of capsaicinoids. This approach preserves the stability and consistency of the product’s pungency.

The organic acid content of NF and IF showed significant dynamic differences during fermentation (Fig. 4G). The initial lactate content of IF was 204.82 mg/100 g, and then increased sharply to 1646.84 mg/100 g on day 5, which was significantly higher than that of NF (589.39 mg/100 g). It indicates that inoculated *L. plantarum* MA1 could greatly promote lactate synthesis. After 20 days of fermentation, the lactic acid contents in IF and NF reached 1771.85 mg/100 g and 1571.61 mg/100 g, respectively, and the formation of lactic acid tended to be stable in the later phase of fermentation. The oxalic acid content in NF increased from 82.95 mg/100 g to 130.60 mg/100 g on day 5, and then fluctuated and decreased to 107.23 mg/100 g on day 20. The oxalic acid content in IF gradually decreased to 77.79 mg/100 g after reaching a peak on day 5, indicating that oxalic acid may be metabolized or converted during the mid-to-late stages of the fermentation process. In addition, the malic acid and succinic acid in NF continued to accumulate during the fermentation process, reaching 165.59 mg/100 g and 270.69 mg/100 g, respectively, by day 20, which were significantly higher than those in IF (81.79 mg/100 g and 58.05 mg/100 g, respectively). The content of acetic acid in IF reached a peak of 653.06 mg/100 g on day 10, which was lower than that in NF. The observed differences indicate that induced fermentation may modulate the fermentation process and the flavor composition of the final product by promoting the early rapid accumulation of lactic acid and influencing intermediate metabolic pathways.

### Characteristic flavor compounds during fermentation of YCS fermented with *L. plantarum*

Volatile compounds in NF and IF groups were analyzed through HS-SPME-GC-MS. A total of 35 volatile compounds were identified, primarily including 11 esters, 9 lipids and lipid-like molecules, 4 alcohols, and 2 benzenoids. Among these, the esters constituted the largest proportion, such as ethyl acetate, hexyl isovalerate, and ethyl 4-methyl valerate.

**Fig. 5:**
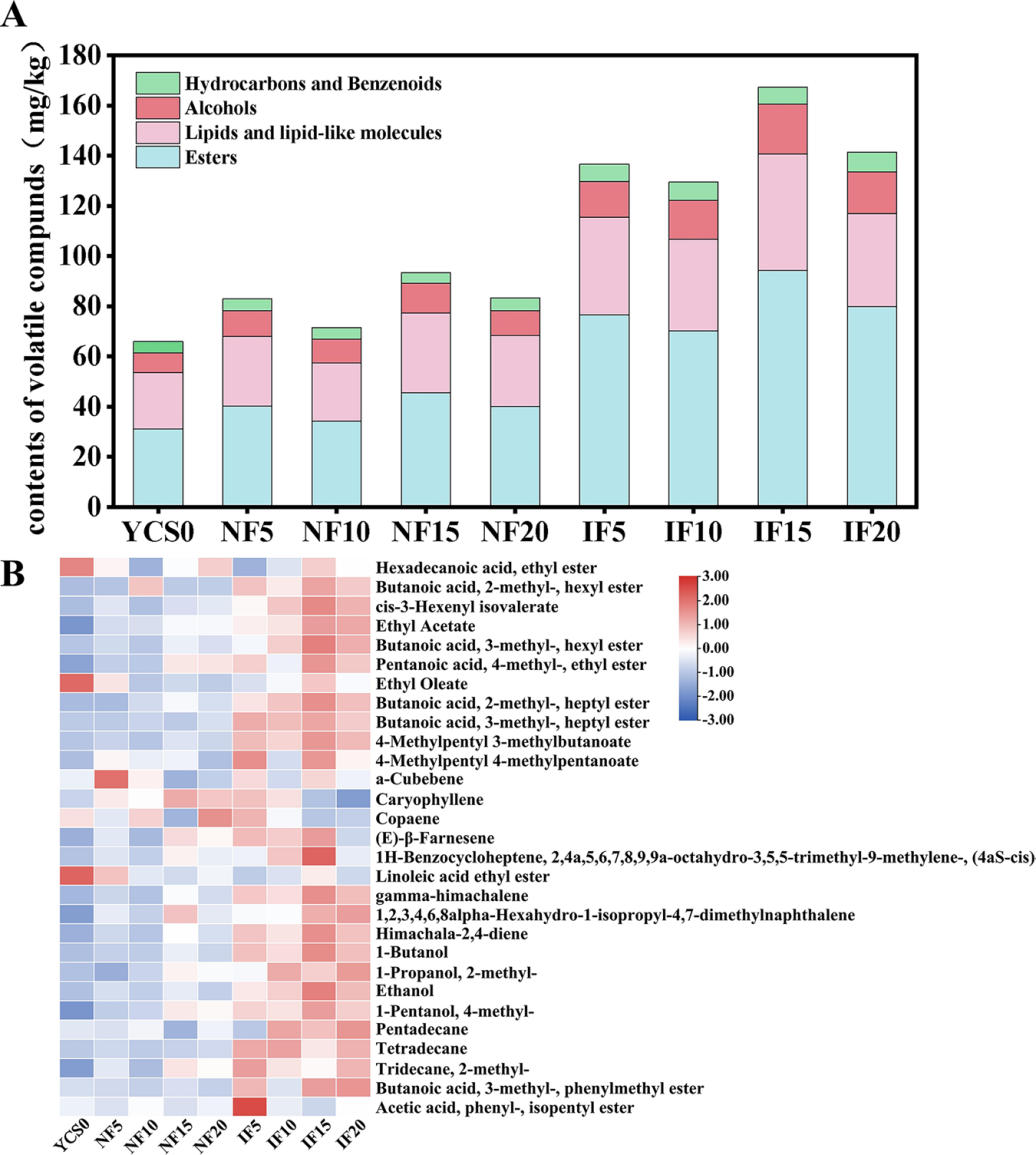
Characteristic flavor compounds during fermentation of YSC fermented with *L. plantarum.* **A** The total content of volatile compounds (mg/kg) in spontaneously fermented (NF) and *Lactiplantibacillus plantarum*-fermented (IF) YCS at different fermentation stages. **B** Changes in volatile components of NF and IF at different fermentation stages. (Source: Microsoft Office PowerPoint, Origin, TBtools).

The contents of VFCs were quantitatively analyzed by the internal standard normalization method (Fig. 5A). The content of VFCs in IF was higher than that in NF, with the increase in esters being particularly notable. For instance, on day 5 of the fermentation process, the ester content in the IF group reached 76.66 mg/kg, which was notably greater than the 40.29 mg/kg in the NF group. Throughout the fermentation process, the total VFC content in IF consistently exceeded that in NF. It peaked on day 15 (167.35 mg/kg), significantly higher than NF (93.48 mg/kg). It is noteworthy that, whether through spontaneous fermentation or induced fermentation, the total VFC content reached its peak on day 15, indicating that this time point may be a critical stage in flavor development.

By comparing the two fermentation methods, it was found that the content of most esters in the inoculation group was generally higher than that in spontaneous fermentation group at all time points. For example, on day 15, the concentrations of *cis*-3-hexenyl isovalerate and hexyl 3-methylbutanoate in IF were 8.64 mg/kg and 9.22 mg/kg, respectively, whereas in NF, corresponding levels were only 3.35 mg/kg and 4.95 mg/kg, respectively (Fig. 5B). As important ester compounds, the synergy between *cis*-3-hexenyl isovalerate and hexyl 3-methylbutanoate formed the sweet fruit aroma. Additionally, the content of ethyl acetate and ethyl 4-methyl valerate in IF was higher than that in NF. The above results show that inoculation with *L. plantarum* MA1 could effectively promote the accumulation of flavor substances in YCS and improve the overall fermentation efficiency.

## Methods

### Sample collection and preparation of spontaneously fermented YCS

**Fig. 6:**
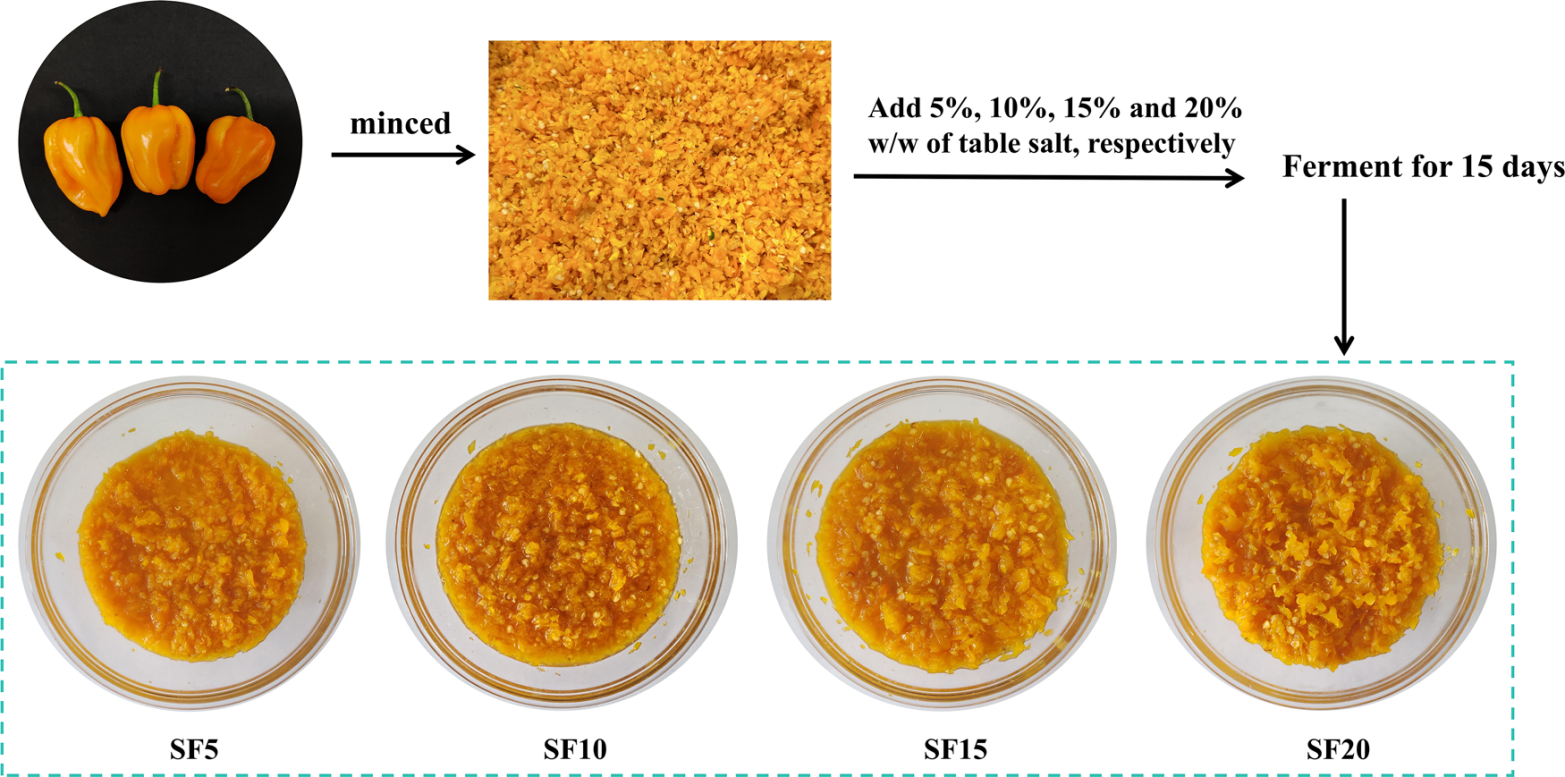
The preparation process of yellow capsicum sauce (YCS). (Source: Photographs taken by the authors, Microsoft Office PowerPoint).

Fresh fruits of *Capsicum chinense* were harvested from the yellow lantern pepper production area in Sanya, Hainan Province, China. After picking, the fruits were rinsed with distilled water and air-dried to remove surface moisture. Subsequently, they were crushed by a crusher (ZG-L74A, Zhigo Holding Co., Ltd.), and 5%, 10%, 15%, and 20% (w/w) of table salt were added respectively (samples were referred to as SF5, SF10, SF15, and SF20). Then, 10% Hainan local rice wine (26% vol) was added and mixed thoroughly. The yellow capsicum sauce was hermetically sealed in a glass jar and underwent fermentation at 30 °C for 15 days (Fig. 6). The sample of YCS was washed with 0.9% (w/v) sterile saline, and the filtrate was collected and centrifuged at 5000 × *g* to obtain the pellet, which was then stored at −80 °C for microbial community analysis.

### Metagenomic analysis of spontaneously fermented YCS

Total DNA was extracted using the ALFA-SEQ DNA Library Prep Kit (Findrop, Guangzhou, China). DNA integrity was assessed by 1% agarose gel electrophoresis, and its concentration and purity were determined using the Qubit 4.0 and NanoDrop One systems (Thermo Fisher Scientific, Waltham, USA). Sequencing was performed on the Illumina NovaSeq 6000 platform at Guangdong Meige Gene Technology Co., Ltd. The 12 samples yielded a total of 1,041.93 million raw reads (156.28 Gb). Individual sample sequencing volumes ranged from 69.35 to 134.93 million reads (10.40–20.24 Gb), with sequencing quality measured at Q30 levels of 96.65–96.92%.

Raw data underwent quality control using fastp, which included the removal of adapter sequences, low-quality reads, and reads shorter than 50 bp, followed by deduplication to mitigate PCR amplification bias. Clean reads were assembled de novo using MEGAHIT (k-min 35, k-max 95, k-step 20). The scaffold was split at consecutive “N” positions to obtain scaftig, retaining only fragments ≥ 500 bp for subsequent analysis. Prodigal was used to predict open reading frames (ORFs), with ORFs < 90 bp removed. Linclust was employed to eliminate redundancy among ORFs and construct a non-redundant gene set.

For taxonomic annotations, the non-redundant Unigenes sequences were blasted for species homology against the NCBI-NR database using BLASTP (Version 2.2.31 + , http://blast.ncbi.nlm.nih.gov/Blast.cgi) with a threshold of e-value ≤ 0.0001. Meanwhile, the predicted protein sequences were subjected to BLAST analysis against the KEGG database (Version: 107.1) to obtain functional annotations. Statistical significance was assessed using a *p*-value < 0.05 to determine the differential abundance in taxonomy and functional annotations.

### Volatile compounds of spontaneously fermented YCS

The volatile compounds were analyzed by headspace solid-phase microextraction method coupled with gas chromatography–mass spectrometry (HS-SPME/GC–MS). Samples (100.0 ± 2.0 mg) were transferred into 20 mL headspace vials, and 10 μL of 2-octanol (10 mg/L) served as the internal standard. The analysis was performed on an Agilent 7890 gas chromatography system equipped with a DB-Wax column (30 m × 250 μm × 0.25 μm) and coupled to a 5977B mass spectrometer. The inlet temperature was set to 50 °C with helium carrier gas at 1.0 mL/min. The initial oven temperature of 40 °C was held for 4 min, then increased to 245 °C at 5 °C/min, and held at the final temperature for 5 min. Mass spectral conditions were 20–400 *m/z*. The solvent delay was 2.13 min^35^. The linear retention index (LRI, with C₆-C₂₄ n-alkanes as references) combined with the NIST20 mass spectrometry database was used for dual qualitative verification, and the semi-quantitative analysis of volatiles was achieved based on the peak area ratio of the target to the internal standard^22^. The corresponding calculation formula is presented below:

1 $$Ci=Cis\times \frac{Ai}{Ais}\times fi$$

In the formula, *Ci* is the corresponding concentration of each volatile component in the sample, mg/kg. *Cis* is the concentration of internal standard, mg/kg. *Ai* is the peak area of each volatile component. *Ais* is the peak area of the internal standard. *fi* is the correction factor.

### The processing and sample collection for YCS fermented with L. plantarum

*L. plantarum* MA1 was isolated from spontaneously fermented YCS (SF5) and preserved in glycerol, stored long-term in our laboratory’s −80 °C freezer. Before the experiment, 0.2 mL of glycerol-preserved *L. plantarum* MA1 strain was transferred into 10 mL of MRS liquid medium and incubated at 37 °C for 24 h. The culture was then sub-cultured to the second generation and incubated at 37 °C for 16–24 h. Subsequently, it was sub-cultured to the third generation and incubated again at 37 °C for 16–24 h. After incubation, the culture was centrifuged at 3,024 × g for 10 minutes, and the bacterial pellet was collected, washed twice with sterile 0.9% (w/v) saline, and then resuspended to a cell density of 10^7^ CFU/mL for inoculation. After crushing the fresh fruits of *Capsicum chinense* with a crusher, 5% (w/w) table salt and 10% (w/w) Hainan traditional rice wine (26% vol) were added, then thoroughly mixed. The yellow capsicum paste was hermetically sealed in a glass jar. The induced fermentation group (IF) was inoculated with 5% a bacterial suspension, while the spontaneous fermentation group (NF) was conducted without inoculation. Both groups were fermented at 30 °C in dark conditions. The fermentation process was monitored, and samples were collected on days 0, 5, 10, 15, and 20. The unfermented YCS (day 0) was labeled as YCS0.

### Acidity and color characteristics analysis of YCS fermented with L. plantarum

The pH value and titratable acidity of fermented peppers were determined according to the Chinese National Standards GB/T 5009.237-2016 and GB 12456-2021, respectively. The color of the YCS was measured using a colorimeter (RC-10, Konica Minolta, Tokyo, Japan). Prior to measurements, the YCS sauce was homogenized. Chromaticity (C*) and Yellowness Index (YI) are computed via the subsequent formula:

2 $$C\ast ={({a}^{\ast 2}+{b}^{\ast 2})}^{0.5}$$

3 $$YI=142.86b\ast /L\ast$$

In the formula, a* indicates the red-green component of the color, with positive values representing red and negative values representing green. b* indicates the yellow-blue component, with positive values representing yellow and negative values representing blue. L* is the lightness of the color (ranging from 0 for black to 100 for white). C* is used to measure the color saturation, with higher C* values indicating more vivid colors. YI is used to measure the yellow intensity of the sample, with higher values indicating a greater yellow intensity in the sample.

### Determination of reducing sugar content of YCS fermented with *L. plantarum*

The reducing sugar content in yellow capsicum sauce was determined by 3,5-dinitrosalicylic acid (DNS) colorimetry^36^. The standard curve for glucose was established with the equation y = 0.5306 x + 0.0484 (R^2^ = 0.9979).

### Determination of nitrite content of YCS fermented with *L. plantarum*

A sample of 2.50 g of the evenly ground YCS sauce sample was placed in a beaker. Then, an aliquot of 6.25 mL of saturated borax solution (50 g/L) was added to the beaker and stirred well. The mixed sample was transferred to a 250 mL volumetric flask containing distilled water and extracted ultrasonically for 30 min. Subsequently, 2.5 mL of potassium ferricyanide solution (106 g/L) and 2.5 mL of zinc acetate solution (220 g/L) were added. The resulting mixture was diluted with distilled water and filtered. The 20-mL filtrate was placed into a 25 mL colorimetric tube, and different volumes of sodium nitrite standard solutions (0.00–2.50 mL, 5.0 μg/mL) were added. An aliquot of 1 mL of sulfanilic acid solution (4 g/L) was added to both the standard tube and the sample tube, followed by mixing and incubation for 5 min. Subsequently, 0.5 mL of naphthalene ethylenediamine hydrochloride solution (2 g/L) was added. The mixture was diluted to 25 mL with distilled water, vigorously shaken, and allowed to stand for 15 min. Finally, the absorbance of both the samples and the standards was determined at 538 nm using a cuvette, and a standard curve for nitrite was constructed.

### Organic acid content of YCS fermented with *L. plantarum*

A sample of 1 g was placed in a centrifuge tube (15–20 mL) and 10 mL of ultrapure water was added. An appropriate amount of the sample was diluted, followed by sonication and centrifugation. The resulting supernatant was collected for subsequent analysis. Analysis was performed using a Hitachi Chromaster High-Performance Liquid Chromatography (HPLC) system, fitted with a photodiode array (PDA) detector and an Agilent ZORBAX SB-Aq column (250 × 4.6 mm, 5 μm). The separation was performed at 30 °C. The mobile phase, composed of perchloric acid solution and methanol (98:2, v/v), was delivered at a flow rate of 0.6 mL/min with an injection volume of 10 μL. Detection was achieved at 210 nm.

### Capsaicin and dihydrocapsaicin content of YCS fermented with *L. plantarum*

The samples were dried at 50 °C in an oven for three days and ground into powder. The dried tissue (1 g) was mixed with ethanol (70%, 50 mL). Subsequently, ultrasonic-assisted extraction was performed to obtain capsaicin and dihydrocapsaicin. A high performance liquid chromatograph (Agilent 1200, Agilent Technologies, USA) was used with a column (250 mm × 4.6 mm, 5 μm, Avantor, New Jersey, USA) at 30 °C and a PDA detector set to 210 nm. Methanol was used as solvent A, and ultrapure water was used as solvent B. The ratio of solvent A to solvent B was 70:30 (v/v), and the flow rate was 1 mL/min. The injection volume was 10 μL. Capsaicin and dihydrocapsaicin standards were used as external standards. Standard stock solutions were prepared and serially diluted to obtain calibration concentrations of 2, 4, 8, 12, 16, and 20 μg/mL. Calibration curves were constructed by plotting peak area versus concentration for quantitative analysis. All measurements were performed in triplicate. The calculation method of Scoville heat units (SHU) is shown in Equation

4 $$SHU=W\times 0.9\times (16.1\times {10}^{3})+W\times 0.1\times (9.3\times {10}^{3})$$

### Aroma compound analysis of YCS fermented with *L. plantarum*

Aroma analysis was performed using an Agilent 6890 N/5973 N GC-MS system, following the method in Section 2.3 with modifications. The sample (3.0 ± 0.1 g) of YCS was placed in a 20-mL headspace vial, and 100 μL of isoamyl phenylacetate (50 μg/mL) was added as an internal standard. Separation was achieved on a HP-INNO Wax column (60 m × 250 μm × 0.25 μm). Helium was used as the carrier gas at a flow rate of 1.5 mL/min. The oven temperature program was as follows: held at 40 °C for 5 min, then ramped at 5 °C/min to 250 °C, and finally held for 10 min.

### Statistical analysis

All statistical analyses were performed based on three independent biological replicate experimental datasets. Statistical analysis was performed using IBM SPSS Statistics 23, with significant differences between groups indicated by letters or asterisks. Data processing and graph plotting for the physicochemical parameters were performed using Origin 2021 GraphPad Prism 9. Multivariate statistical analysis was carried out using SIMCA 14.1. A Correlation network diagram and a heat map were plotted with Cytoscape and TBtools, respectively.

## Supplementary information

Supplementary material

## Data Availability

The sequence data reported in this study have been deposited in the NCBI database under BioProject ID PRJNA1268923. All other data supporting the findings of this study are available within the Article and its Supplementary Information files or from the corresponding authors upon reasonable request.
