# Supplementary material for: Lactiplantibacillus plantarum-mediated modulation of volatile flavor and quality in low-salt spontaneously fermented yellow capsicum sauce
Source: NPJ Sci Food. 2026 Apr 22;10:204. doi: 10.1038/s41538-026-00854-z (PMC13315914; doi:10.1038/s41538-026-00854-z)
Supplement: Supplementary file 1 — Supplementary material [file 41538_2026_854_MOESM1_ESM.doc]

Table .S1 Volatile compounds identification and flavor description of Yellow capsicum sauce with different salt contents.

| Component Name | Molecular Formula | RIcal | CAS | Odour threshold（mg/kg）in water | concentration（mg/kg） | | | | Odorant descripyion |
| --- | --- | --- | --- | --- | --- | --- | --- | --- | --- |
|  |  |  |  |  | SF5 | SF10 | SF15 | SF20 |  |
| **Alcohols** |  |  |  |  |  |  |  |  |  |
| Ethanol | C2H6O | 924.7783 | 64-17-5 | 950 | 496.20±103.05 | 425.98±58.33 | 401.12±69.75 | 362.07±57.82 | / |
| 1-Propanol | C3H8O | 1029.5182 | 71-23-8 | 8.5056 | 0.26±0.09 | 0.23±0.04 | 0.18±0.03 | 0.15±0.03 | Alcohol, Candy, Pungent |
| 1-Butanol | C4H10O | 1137.5583 | 71-36-3 | 0.4592 | 0.32±0.07 | 0.32±0.05 | 0.24±0.02 | 0.20±0.04 | Fruit |
| 1-Penten-3-ol | C5H10O | 1151.1 | 616-25-1 | 0.3581 | 0.25±0.03 | 0.29±0.05 | 0.21±0.04 | 0.22±0.06 | Butter, Fish, Green, Oxidized, Wet Earth |
| 1-Butanol, 2-methyl- | C5H12O | 1196.9833 | 137-32-6 | 0.0159 | 3.28±1.88 | 2.51±0.45 | 2.55±0.76 | 1.13±0.34 | Fish Oil, Green, Malt, Onion, Wine |
| 1-Butanol, 3-methyl- | C5H12O | 1198.26 | 123-51-3 | 0.291 | 6.12±3.03 | 4.93±0.79 | 4.87±1.17 | 2.40±0.71 | Burnt, Cocoa, Floral, Malt |
| 1-Pentanol | C5H12O | 1239.01 | 71-41-0 | 0.1502 | 4.02±0.89 | 4.19±0.53 | 3.18±0.92 | 2.67±0.67 | Balsamic, Fruit, Green, Pungent, Yeast |
| 2-Heptanol | C7H16O | 1307.05 | 543-49-7 | 0.065235 | 1.34±0.64 | 0.96±0.22 | 1.03±0.35 | 0.28±0.11 | Citrus, Earth, Fried, Mushroom, Oil |
| 1-Hexanol | C6H14O | 1338.5167 | 111-27-3 | 0.0056 | 36.97±6.72 | 40.66±6.24 | 30.47±7.30 | 21.04±6.45 | Banana, Flower, Grass, Herb |
| 3-Hexen-1-ol, (E)- | C6H12O | 1366.2667 | 928-97-2 | 0.11 | 21.43±5.06 | 26.51±2.93 | 17.84±5.70 | 14.89±3.85 | Green |
| 1-Heptanol | C7H16O | 1437.0909 | 111-70-6 | 0.0054 | 1.80±0.44 | 2.34±0.52 | 1.86±0.62 | 1.28±0.36 | / |
| 1-Octanol | C8H18O | 1536.0091 | 111-87-5 | 0.1258 | 0.08±0.01 | 0.10±0.04 | 0.09±0.01 | 0.06±0.01 | Bitter Almond, Burnt Matches, Fat, Floral |
| 2-Furanmethanol | C5H6O2 | 1632.4 | 98-00-0 | 4.5005 | 0.78±0.60 | 0.10±0.02 | 0.69±0.53 | 0.06±0.02 | Burnt, Caramel, Cooked |
| Benzeneacetic acid, hexyl ester | C14H20O2 | 2095.65 | 5421-17-0 | / | 0.22±0.05 | 0.14±0.07 | 0.20±0.05 | 0.25±0.12 | Fruit |
| 1-Hexadecanol | C16H34O | 2354.6 | 36653-82-4 | / | 0.04±0.01 | 0.07±0.01 | 0.06±0.01 | 0.08±0.01 | Flower, Wax |
| **Esters** |  |  |  |  |  |  |  |  |  |
| Ethyl Acetate | C4H8O2 | 889.6008 | 141-78-6 | 0.005 | 8.01±7.62 | 0.73±0.12 | 5.28±5.04 | 0.20±0.06 | Aromatic, Brandy, Grape |
| Propanoic acid, 2-methyl-, ethyl ester | C6H12O2 | 952.82 | 97-62-1 | 0.00002 | 0.42±0.22 | 0.34±0.07 | 0.30±0.12 | 0.38±0.19 | / |
| Butanoic acid, ethyl ester | C6H12O2 | 1025.45 | 105-54-4 | 0.009 | 0.13±0.08 | 0.08±0.02 | 0.08±0.02 | 0.09±0.04 | Apple, Butter, Cheese, Pineapple, Strawberry |
| Butanoic acid, 2-methyl-, ethyl ester | C7H14O2 | 1040.8583 | 7452-79-1 | 0.000063 | 3.52±1.57 | 4.15±0.79 | 3.15±2.36 | 3.09±1.69 | Apple, Ester, Green Apple, Kiwi, Strawberry |
| Butanoic acid, 3-methyl-, ethyl ester | C7H14O2 | 1055.3083 | 108-64-5 | 0.00001 | 10.84±4.89 | 15.35±2.62 | 9.86±7.45 | 8.75±4.66 | Apple, Fruit, Pineapple, Sour |
| Pentanoic acid, ethyl ester | C7H14O2 | 1119.025 | 539-82-2 | 0.0058 | 0.29±0.12 | 0.24±0.06 | 0.23±0.11 | 0.23±0.10 | Apple, Dry Fish, Herb, Nut, Yeast |
| Pentanoic acid, 4-methyl-, ethyl ester | C8H16O2 | 1175.9667 | 25415-67-2 | 0.000003 | 14.20±4.36 | 12.28±2.45 | 11.57±5.68 | 9.35±3.95 | / |
| Acetic acid, hexyl ester | C8H16O2 | 1214.7364 | 142-92-7 | / | 1.21±0.61 | 0.66±0.19 | 0.88±0.35 | 0.34±0.15 | Apple, Banana, Grass, Herb, Pear |
| Hexanoic acid, ethyl ester | C8H16O2 | 1218.6417 | 123-66-0 | 0.005 | 2.52±0.11 | 2.62±0.94 | 1.81±0.69 | 1.53±0.46 | Apple Peel, Brandy, Fruit Gum, Overripe Fruit, Pineapple |
| 3-Hexenoic acid, ethyl ester | C8H14O2 | 1296.525 | 2396-83-0 | / | 0.88±0.28 | 0.61±0.21 | 0.76±0.24 | 0.59±0.32 | Fruit |
| Butanoic acid, 3-hydroxy-, ethyl ester | C6H12O3 | 1488.575 | 5405-41-4 | 40-50 | 0.32±0.09 | 0.28±0.04 | 0.27±0.04 | 0.17±0.04 | Marshmallow, Roasted Nut |
| Hexanoic acid, hexyl ester | C12H24O2 | 1593 | 6378-65-0 | 6.4 | 0.02±0.02 | 0.01±0.01 | 0.00±0.00 | 0.00±0.00 | Apple Peel, Peach, Plum |
| Benzoic acid, 2-methylpropyl ester | C11H14O2 | 1768.95 | 120-50-3 | / | 0.07±0.01 | 0.09±0.03 | 0.06±0.03 | 0.05±0.01 | Fruit |
| Octanoic acid, hexyl ester | C14H28O2 | 1801.25 | 1117-55-1 | / | 0.04±0.00 | 0.04±0.01 | 0.03±0.01 | 0.04±0.02 | Green, Herb, Oil |
| Nonanoic acid, hexyl ester | C15H30O2 | 1810.1 | 6561-39-3 | / | 0.05±0.02 | 0.25±0.33 | 0.06±0.03 | 0.11±0.05 | Fruit |
| Acetic acid, decyl ester | C12H24O2 | 2001.4 | 112-17-4 | 0.225-1 | ND | 0.02±0.00 | 0.01±0.01 | 0.02±0.01 | Oil, Orange |
| Ethyl Oleate | C20H38O2 | 2441.8583 | 111-62-6 | / | 0.18±0.02 | 0.27±0.03 | 0.22±0.03 | 0.32±0.04 | Dairy |
| Propanoic acid, 2-oxo-, ethyl ester | C5H8O3 | 1248.92 | 617-35-6 | / | 0.01±0.00 | 0.02±0.01 | ND | 0.01±0.00 | Floral |
| **Aldehydes** |  |  |  |  |  |  |  |  |  |
| Pentanal | C5H10O | 962.6 | 110-62-3 | 0.012 | 0.14±0.03 | 0.22±0.05 | 0.18±0.01 | 0.13±0.05 | Almond, Bitter, Malt, Oil, Pungent |
| Hexanal | C6H12O | 1064.3 | 66-25-1 | 0.005 | 0.10±0.07 | 0.10±0.03 | 0.06±0.02 | 0.05±0.01 | Apple, Fat, Fresh, Green, Oil |
| 2-Butenal, 2-methyl- | C5H8O | 1073.2 | 1115-11-3 | 0.5 | 0.08±0.06 | 0.08±0.02 | 0.07±0.06 | ND | / |
| Heptanal | C7H14O | 1166.5727 | 111-71-7 | 0.0028 | 0.05±0.01 | 0.04±0.01 | 0.03±0.00 | 0.02±0.00 | Citrus, Fat, Green, Nut |
| Nonanal | C9H18O | 1373.64 | 124-19-6 | 0.0011 | 0.10±0.01 | 0.06±0.01 | 0.08±0.04 | 0.09±0.03 | Fat, Floral, Green, Lemon |
| **Ketones** |  |  |  |  |  |  |  |  |  |
| 3-Penten-2-one | C5H8O | 1108.1364 | 625-33-2 | 1.2 | 0.44±0.08 | 0.23±0.08 | 0.32±0.06 | 0.24±0.12 | / |
| 2-Heptanone | C7H14O | 1164.9455 | 110-43-0 | 0.14 | 0.66±0.36 | 0.55±0.18 | 0.42±0.11 | 0.10±0.04 | Blue Cheese, Fruit, Green, Nut, Spice |
| 3-Octanone | C8H16O | 1236.1125 | 106-68-3 | 0.0214 | 0.02±0.00 | 0.03±0.01 | 0.02±0.00 | ND | Butter, Herb, Mold |
| Acetoin | C4H8O2 | 1260.0444 | 513-86-0 | 0.014 | 0.42±0.27 | 2.48±0.70 | 0.40±0.06 | 0.20±0.08 | Butter, Creamy, Green Pepper |
| 2-Butanone, 4-(2,6,6-trimethyl-1-cyclohexen-1-yl)- | C13H22O | 1814.7 | 17283-81-7 | 0.001 | 0.21±0.02 | 0.21±0.06 | 0.17±0.04 | 0.14±0.04 | Floral |
| 5,9-Undecadien-2-one, 6,10-dimethyl- | C13H22O | 1841.1909 | 689-67-8 | / | 0.09±0.02 | 0.12±0.03 | 0.09±0.02 | 0.11±0.04 | Fruit |
| **Acids** |  |  |  |  |  |  |  |  |  |
| Propanoic acid, 2-methyl- | C4H8O2 | 1542.23 | 79-31-2 | 6.5505 | 0.03±0.01 | 0.05±0.01 | 0.02±0.01 | 0.03±0.01 | Burnt, Butter, Cheese, Sweat |
| 2-Pentenoic acid, 2-methyl- | C6H10O2 | 1948.3182 | 3142-72-1 | / | 0.10±0.03 | 0.10±0.01 | 0.08±0.01 | 0.11±0.00 | Fruit |
| **Alkanes,terpenes** |  |  |  |  |  |  |  |  |  |
| Hexane, 3,3-dimethyl- | C8H18 | 996.91 | 563-16-6 | / | 0.04±0.01 | 0.03±0.01 | 0.03±0.00 | 0.02±0.00 | / |
| Caryophyllene | C15H24 | 1569.4455 | 87-44-5 | 0.064 | 0.42±0.11 | 0.47±0.20 | 0.38±0.11 | 0.45±0.16 | Fried, Spice, Wood |

Note: Results are expressed as mean values with standard deviation. “/” means no references are available. “ND” means not detected.





# Fig.S1: The SHU of spontaneously fermented (NF) and *Lactiplantibacillus plantarum*-fermented (IF). (Soure: Origin)
